# Causes and consequences of adolescent dating violence: a systematic review

**DOI:** 10.5249/jivr.v11i2.1061

**Published:** 2019-07

**Authors:** Stella R. Taquette, Denise Leite Maia Monteiro

**Affiliations:** ^*a*^ Department of Pediatrics, State University of Rio de Janeiro, Brazil.; ^*b*^ Department of Obstetrics and Gynecology, State University of Rio de Janeiro, Brazil.

**Keywords:** Dating violence, Gender, Adolescence, Exposure to violence

## Abstract

**Background:**

Adolescent dating violence (ADV) is highly prevalent and can have serious health consequences, including homicides, and be a predictor of intimate partner violence in adulthood. This review aims to systematize the knowledge produced in recent empirical investigations in health that focus on the causes and consequences of ADV to subsidize new research and prevention programs.

**Methods:**

Review of studies published in PubMed over the last five years through MeSH Database: “Intimate Partner Violence” AND “Adolescent” NOT “prevention and control” NOT “Adult”.

**Results:**

We analyzed 35 papers, of which 71.4% were developed in the USA. Some studies have shown prevalence greater than 50% in both genders, both as victims and perpetrators, with more serious consequences for females. Three main thematic cores were identified in the studies: ADV-related vulnerabilities, circularity of violence and ADV-associated health problems. Data indicate that ADV is deep-seated in the patriarchal culture and is more frequent in connection with racism, heterosexism and poverty. It occurs in a circular way and is linked to other forms of violence in different contexts (family, school, community and social media). It is associated with health problems such as depression, anxiety, low self-esteem, alcohol and drugs abuse and unprotected sex.

**Conclusions:**

The knowledge produced in the studies reviewed reveals the urgency and importance of implementing early preventive actions in schools, involving families and the community. These should focus on the deconstruction of current cultural gender patterns, based on their historical origin, in order to support emancipatory and liberating pedagogical approaches.

## Introduction

Adolescent Dating Violence (ADV) is defined as any intentional, psychological/emotional, physical or sexual abuse that occurs between people involved in a romantic relationship.^1^ It is a significant event in several parts of the world^2^ and can have immediate and late health consequences, and women are the most serious and frequent injury victims.^3,4^ ADV is often not perceived by those involved or valued by society, although it may culminate in murders, usually of women, as in abusive relationships in adulthood.^5^ ADV is a predictor of marital violence in adulthood.^5^ Femicide in adulthood has been addressed as an important public health problem and has been gaining prominence also concerning the health of adolescents.^6,7^

Several population-based studies have shown health problems associated with ADV, including depression, anxiety, and alcohol and other drugs abuse.^8^ Other authors point out the sexual risk behavior of STI/AIDS and the low academic performance as negative consequences of ADV.^9^ In addition, there is evidence that violence in other settings, such as in the family and neighborhood may be related to ADV.^8^

ADV is a health problem in different parts of the world and one of the main issues to be tackled.^7^ Consequently, several policies and action programs have been implemented, especially in the school setting, the main arena for socialization and construction of adolescents’ identity.^10,11^ However, the issue is hardly discussed in developing countries, and knowledge about the subject is still incipient, hampering sensitization of people about the problem and policy formulation to address it.^12^

This bibliographic review is being proposed considering the challenges faced in addressing situations of violence involving intimate adolescents and the recognition of the need to act early to prevent this type of violence. It aims to systematize the knowledge produced in empirical investigations in the field of health focused on the causes and consequences of ADV. This review intends to subsidize new research and prevention programs that contribute to curb violence in intimate adolescent relationships.

## Methods 

This is a review of studies published in PubMed, the most important database of health studies. This database was chosen because it is the one that gathers the largest number of bibliographical sources from around the world in the field of health and journals of recognized quality. In general, papers published in qualified journals from other databases are also found in PubMed.

The following search strategy was followed on June 28, 2017 through MeSH Database: “Intimate Partner Violence” AND “Adolescent” NOT “prevention and control” NOT “Adult” in the last five years. Most of the articles found with this search strategy predominantly use the terminology Dating Violence. 

**Figure 1:**
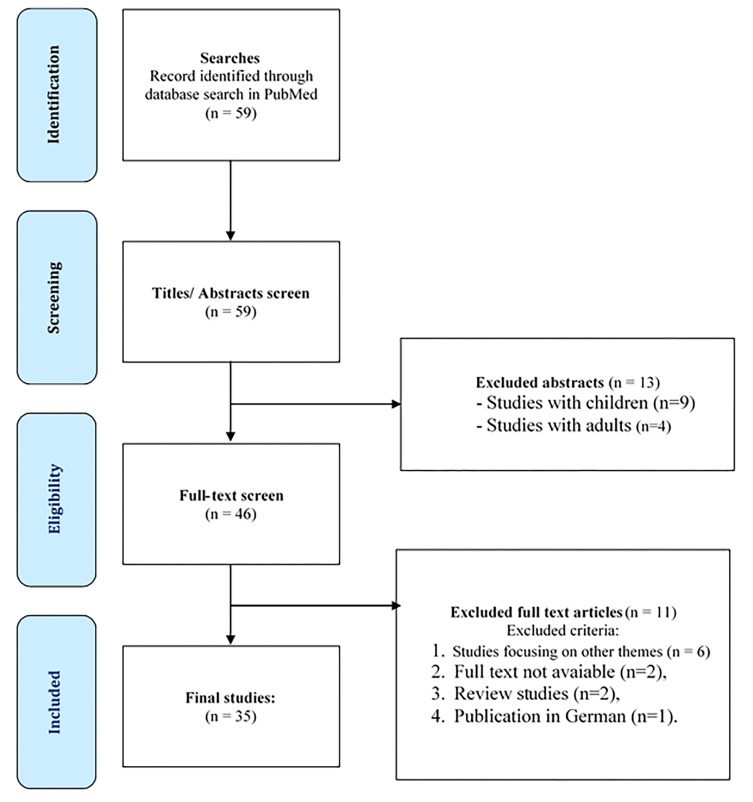
Study selection chart. Adapted from the PRISMA group 2009 flowchart.

Fifty-nine papers were made available. Then, after reading abstracts, 13 papers were excluded from the established inclusion criteria – papers with full-text in English, derived from empirical research with adolescents on ADV: 9 because they were studies with children and 4 with adults. After further reading, eleven more papers were excluded: 6 because they were not on the subject of the study, 2 because the full-texts were not available, 2 because they were review papers and 1 because they were published in German. Thus, a total of 35 papers were analyzed, as can be seen in the chart below (Figure 1).

The thematic analysis of papers was carried out. First, separately by each author. Subsequently, authors showed each other’s analyses and reached the final categories through dialogue with literature. The following steps marked by Minayo13 were followed: comprehension reading and rereading to familiarize with data; identification of themes of analysis considering the objectives of the study; classification of themes and comparative dialogue with literature; and elaboration of interpretative synthesis.

In addition to papers of this review shown in Table 1,2 and 3 in the results, other 19 papers included in the introduction to the text and in data discussion were read.

**Table 1:** Revised papers that gave rise primarily to category I.

AUTHOR/YEAR/DESIGN/LOCAL	CAT	OBJECTIVES	SAMPLE	RESULTS/CONCLUSIONS
14 Shamu S et al., 2016, cross-sectional, South Africa –(Pretoria)	I	To investigate the prevalence of and factors associated with girls’ experience and boys’ perpetration of Intimate IPV	3,755 grade 8 learners	The prevalence of IPV experience by girls was 30.9% and perpetration by boys was 39.5%. The factors associated with girls' experience of IPV include childhood violence, gender inequitable attitudes, and corporal punishment, among others.
18 Pearlman DN, Dunn HK, 2016, cross-sectional, USA (Rhode Island)	I, II, III	To examine overlapping forms of peer and dating abuse from a gendered perspective	6,390 students from 9th to 12th grades	Almost half of the students reported peer two or more forms of violence. Bullying was the most prevalent form of abuse (97.1%), followed by homophobic teasing (52.7%) and teen dating violence (40.9%).
19 Reidy DE, et al, 2016, cross-sectional, USA (Texas)	I	To assess gender differences of adolescents reporting TDV and the frequency of TDV at multiple age points	1,149 teens ages 11 to 17 years	The data suggest that at specific times during adolescence, boys among high-risk populations may be equally at risk for victimization. However, the psychological consequences (fear) are greater for girls.
20 Calvete E et al, 2016, cohort. Spain (Bizkaia)	I, II	To assess the role of the social information processing (SIP) in dating aggression.	1,272 secondary students (653 girls, 619 boys)	Girls presented higher rates of psychological aggression, whereas boys presented higher rates of sexual aggression. Hostile attribution, anger and aggressive response access are correlated factors in TDV.
21 Diaz-Aguado MJ, Martinez R, 2015, cross-sectional, Spain	I	To establish a typology of male adolescents to contribute to prevent gender violence	4,147 boys aged 14 to 18 years	4 groups were identified: non-violent adolescent boys, boys who isolate and control their partners; boys who exert medium-level of abuse and boys who frequently engage in all types of violence.
22 Reidy DE et al, 2015, cross-sectional, USA (Michigan)	I	To assess self-perceptions of gender role discrepancy and history of TDV.	589 male adolescents	Boys who experience stress about being perceived as "sub-masculine" may be more likely to engage in sexual violence as a means of demonstrating their masculinity to self and/or others.
23 Gressard LA et al, 2015, cross-sectional, USA.	I	To determine whether the gender inequality index (GII) correlates with levels of TDV victimization.	413,583 high school students.	The prevalence of physical TDV victimization ranged from 7.0% to 14.8%, and the prevalence of sexual ADV victimization ranged from 7.8% to 13.8%. The GII was significantly associated with the state prevalence of female physical ADV victimization.
24 Coker AL et al, 2014, cross-sectional, USA (Kentucky)	I, II	To estimate the prevalence rates of TDV by demographic factors and other forms of interpersonal violence	14,190 high school students	Rates of DV victimization and perpetration were highest among females, those receiving free or reduced-price meals, those not exclusively attracted to the opposite sex, students reporting parental or guardian partner violence, binge drinking, and bullying.
25 Taylor BG, Mumford EA., 2016, cross-sectional, USA	I	To examine the national prevalence rate of adolescent relationship abuse (ARA)	1,804 teens – 12 to 18-years old	69% of respondents reported lifetime ARA victimization and 63% perpetration. Psychological abuse was the most common and 12% reported perpetration physical abuse and/or sexual abuse. Gender differences were observed.
26 Messinger AM et al, 2014, cross-sectional, USA (New York)	I	To test in adolescents the Johnson's intimate partner violence (IPV) typology in adults	493 female high school students	The results suggest that the pattern of adolescent IPV differs substantially from that of adult IPV and that a relationship-level typology provided additional clarity in categorizing this pattern.
29 Mahmoud AM et al, 2016, cross-sectional, Egypt	I	To assess the knowledge, attitudes and practices of adolescents in Upper Egypt on domestic gender-based violence-GBV	400 boys and girls aged 11-16 years	The proportion of adolescents who could identify certain practices as forms of GBV was low. 65.6% of study participants could correctly identify the legal age of marriage as 18 years, yet only 22.0% identified earlier ages of marriage as a form of domestic GBV.
30 Nagamatsu M et al, 2016, cross sectional, Japan	I	To investigate factors associated with the ability of students to recognize dating violence	3,050 students aged from 13 to 15 years	Boys and girls with more knowledge of dating violence, who focused on an equal dating relationship showed a greater ability to recognize the signs of dating violence.
34 Ahonen L, Loeber R.2016, cohort, USA (Pittsburg)	I, II	To investigate possible underlying factors for involvement in TDV* either as a perpetrator or a victim	2,450 female adolescents	Results show a moderately strong association between parents' negative emotion regulation and their daughters' involvement in serious dating violence.
35 Boafo IM, et al, 2014, cross-sectional, South Africa (Capetown)	I	To examine the relationship between TDV and self-efficacy for delayed sex	3,655 students aged 12 to 17 years	The result revealed that there was a significant association between self-efficacy for delayed sex and socio-economic status, but this link decreased with age.
36 Luo F et al, 2014, cohort, USA	I	To examine whether sexual minority youths (SMYS) are at increased risk for TDV	62,861 adolescents	SMYs have significantly increased odds of TDV compared with non-SMYs.
37 Martin-Storey A, 2015, cross-sectional, USA (Massachusetts)	I	To assess the prevalence of TDV of sexual minority status	12,984 adolescents ages 14-18 years	The results supported a higher prevalence of dating violence among sexual minority youth. This vulnerability varied considerably across gender, sexual minority identity and the gender of sexual partners, but generally persisted when accounting for the mediating variables.

*TDV: Teen Dating Violence; ** IPV: Intimate Partner Violence; *** RR: Relative Risk

**Table 2:** Revised papers that mainly gave rise to category II.

AUTHOR/YEAR/DESIGN/LOCAL	CAT	OBJECTIVES	SAMPLE	RESULTS/CONCLUSIONS
15 Foshee VA et al, 2016, cross-sectional, USA (North Caroline)	II	To test risk factors for the TDV, bullying, and sexual harassment (SH) among adolescents who had been exposed to domestic violence	399 mother and their adolescents	70 % of the adolescents reported perpetrating at least one of the 3 forms of aggression. Poor conflict management skills was a risk for bullying and SH, but not TDV; acceptance of dating violence was a risk for dating violence and bullying, but not SH.
16 Roman NV, Frantz JM., 2013, cross-sectional, Africa (7 countries)	II	To establish the prevalence of IPV and the implications of exposure on adolescents in Africa	7 epidemiological studies	The prevalence of IPV in African countries ranged from approximately 26.5% to 48%. All studies reported exposure to family violence during childhood.
19 Reidy DE et al 2016a, cross-sectional, USA (Texas)	II	To identify distinct classes of adolescents that commit TDV and assess differences on behaviors	1,149 adolescents with violence exposure	The largest class of students was nonviolent on all indices ("nonaggressors") and the smallest class of students demonstrated high probability of nearly all indices of TDV ("multiform aggressors").
27 Aho N et al, 2016, cross-sectional, Sweden	II, I	To measure the prevalence of victimizing events and of poly-victimization	5,960 high schools students (aged 17)	84.1% of the students had experienced victimization during their lifetime, and 10.3% were categorized as poly-victims.
33 Earnest AA, Brady SS., 2016, cross-sectional, USA (Minnesota)	II, I	To examine whether being a victim of violence in the household and feeling unsafe at school are associated with TDV	75,590 ninth-and twelfth grade students	Significant differences were found by gender, grade, ethnicity, and free/reduced price lunch status. Being a victim of violence in the household and feeling unsafe at school, and low perceived care by parents were strongly associated with dating violence victimization.
38 Copp JE et al, 2015, cross-sectional. USA (Ohio)	II, III	To examine the role of anger and depression in the association between neighborhood disadvantage and IPV.	1,321 students	The anger and depressive symptoms partially explain the association between neighborhood disadvantage and IPV. The associations between disadvantage, disorder, and IPV depend on respondent's level of anger.
39 Ellis WE, Wolfe DA, 2015, cross-sectional, Canada	II	To examine the relationship between reported bullying and TDV	585 teens, with dating experience	Bullying positively predicted dating violence perpetration and victimization. Self-reported bullying also predicted observations of lower relationship support and higher withdrawal.
41 Reyes HL et al , 2015, cohort. USA.	II	To examined whether social control and violence in other contexts moderate the associations between substance abuse and TDV	1,920 students	Physical dating violence perpetration increased at time points when heavy alcohol and hard drug use were elevated; these associations were weaker when neighborhood social control was higher and stronger when family violence was higher.
42 Viejo C et al, 2016, cross-sectional, Spain and United Kingdom	II	To examine and compare the prevalence and characteristics of physical dating violence among young people in England and Spain	England (199) and Spain (200) teens aged 15 to 18	Approximately 23% of young people reported victimization and 30% reported perpetrating physical dating violence. In both countries, most of those involved in physical dating violence reported involvement in reciprocal violence.
43 Cascardi M., 2016, cohort, USA.	II	To examine whether psychological distress mediated the violence in childhood and early adolescence and TDV in young adulthood	532 female adolescents	Psychological distress may play a causal role in the relationship of violence in the home to TDV. Interventions targeting psychological distress, particularly in samples at risk for child maltreatment, may reduce the risk of dating violence victimization.
44 Temple JR et al, 2016a, cohort, USA (Texas)	II, III	To examine whether abuse perpetration mediates the acceptance of dating violence and mental health	1,042 ethnically diverse students	Acceptance of dating violence is a risk factor for negative psychological outcomes among adolescents who perpetrate psychological abuse
45 Mumford EA et al, 2016, cohort, USA.	II	To investigate whether there are distinguishable parenting profiles with youth's attitudes about abusive dating behavior	1,117 parent-youth dyads (ages 12-18 years)	Youth in the "Positive Parenting" class were significantly less likely 1 year later to be tolerant of violence against boyfriends under any conditions as well as less likely to perpetrate adolescent relationship abuse or to be a victim of adolescent relationship abuse.
46 Temple JR, et al., 2016b, cohort, USA (Texas)	II	To examine whether dating abuse in one context predicts cyber dating abuse.	780 adolescents(58% female)	Traditional and cyber abuse were positively associated. Cyber abuse perpetration in the previous year predicted cyber abuse perpetration 1 year later.

* TDV: Teen Dating Violence; ** IPV: Intimate Partner Violence; *** RR: Relative Risk

**Table 3:** Revised papers that mainly gave rise to category III.

AUTHOR/YEAR/DESIGN/LOCAL	CAT	OBJECTIVES	SAMPLE	RESULTS/CONCLUSIONS
32 Alleyne-Green B et al, 2016, cross-sectional, USA	III, II, I	To explored the relationship between of biological fathers and the sexual risk behaviors and dating violence of adolescent girls	879 female adolescents	The more TDV an adolescent girl experiences, the less likely she is engaged in healthy sexual behaviors. TDV was directly associated with risky sexual behaviors among sexual experimented adolescents girls, particularly non-White girls.
50 Martz DM et al, 2016, cross sectional. USA (North Caroline)	III	To examine the association between physical and sexual IPV** and other risk factors	1,003 high school students	Each form of IPV was associated with greater risk for depression and suicidal behaviors, substance use, risky sexual behaviors. RR*** tended to be more robust and statistically significant for females compared with males.
51 Whisman MA, et al., 2014, cross-sectional, USA	III	To examine the associations between intimate relationship involvement, intimate relationship quality, and psychiatric disorders	1,566 adolescents in a serious intimate relationship	The prevalence of mood, anxiety, substance use disorders, and several specific disorders were significantly associated with (a) being married or involved in a serious relationship; and (b) reporting more negative relationship quality.
52 Zaha R et al, 2013, cross-sectional, USA (Hawaii)	III	To explored the relationship between adolescent substance use and intimate partner violence (IPV).	4,364 public school students	IPV victimization and substance use are prevalent among Hawai'i youth. Odds ratio calculations indicated that substance use is associated with an increased likelihood of reporting IPV victimization.
53 McNaughton Reyes HL et al, 2014, cohort, USA (North Caroline)	III	To examine proximal and time-varying relations between drug use and physical dating aggression	2,455 students attending	Proximal effects of marijuana use on dating aggression were found for girls and proximal effects of hard drug use on dating aggression were found for boys.
54 Baker CK., 2016, qualitative analisys, USA (Hawaii)	III	To examine the context in which occur two health associated problems: adolescent dating violence (ADV) and substance use	8 sex-specific focus groups with 39 high school students	Adolescents use alcohol and/or drugs at the start of the dating relationship and after the relationship ended as a way to cope with the breakup. Alcohol and drugs were also used throughout to cope with being in an abusive relationship.

*TDV: Teen Dating Violence; ** IPV: Intimate Partner Violence; *** RR: Relative Risk

## Results and Discussion

Most of the studies used a quantitative method, of which 25 (71.4%) were cross-sectional studies and nine (25.7%) were longitudinal cohort studies. Only one (2.8%) of the studies used a qualitative method using focal groups and participant observation. As for the location, 26 were conducted in North America (25 in the U.S. and one in Canada); four in Africa (1 in Egypt, 2 in South Africa and 1 in several countries - Egypt, South Africa, Liberia, Kenya, Malawi, Rwanda and Zambia); 4 in Europe (1 UK, 2 Spain and 1 Sweden); and 1 in Asia (Japan).

ADV prevalence rates were variable, with the highest one found at 70.7%, in a study carried out in Egypt with 400 adolescents of both genders, referring to any type of ADV, whether psychological, physical or sexual.^14^ The second highest rate was 70%, shown in a research developed in the U.S. with 399 adolescents.^15^ A study conducted in seven African capitals evidenced ADV prevalence ranging from 26.5% to 48%.^16^ In other studies with a higher number of adolescents, rates were lower: 33.4% in a study of 14,190 secondary school students in the U.S.^17^ and 27.7% in a sample of 6,390 American adolescents.^18^ It is worth noting that there are different types of dating-related abuse and that, according to Reidy’s study,^19^ few adolescents engage in multiple forms of violence and most young people are not perpetrators.

Considering the aim of this review to highlight the causes and consequences of ADV, three main thematic cores were identified in the studies: I– ADV-related vulnerabilities; II– circularity of violence; and III– ADV-associated health problems. We sorted papers in three tables, in a didactic way according to the main thematic core that originated them, although papers identified drew characteristics of more than one thematic core or all, as described and analyzed below. Table 1,2 and 3 show the study design, location, thematic categories, objectives, sample studied and results/conclusions. They are shown at the end of each category.

**I - ADV-related vulnerabilities**

The concept of vulnerability applied to health emerged and gained momentum in the 1990s in the face of the HIV/AIDS epidemic.^20^ It refers to a set of aspects, not only individual, but also collective and social that lead to greater susceptibility to illness. In the analysis of papers in this bibliographic review, we observed that certain vulnerabilities, similar to those observed in the HIV/AIDS epidemic, are identified as ADV-related, including gender inequality, low age, racial discrimination, homophobia and poverty.

Among these factors, what stands out the most in the reviewed works is gender inequality. This feature underpins the patriarchal culture, in which the social roles played by men and women are assigned different powers. Men are domineering and women dominated, which makes men naturally aggressive and strong, and women, in turn, fragile and helpless. Oftentimes, this domination relationship that justifies violence in certain situations is not perceived even in cases of physical abuse that are sometimes considered normal in a dating relationship.^3^

The patriarchal culture favors the boys’ perpetration of violence, because when they feel their virilities threatened, they try to impose themselves by violent means and consider this violence of lesser importance.^21^ Thus, gender inequality is a risk factor for violence against women in this age group.^22,23^

The revised papers reveal that both boys and girls are ADV victims and perpetrators, with prevalence varying according to the stage of adolescence. In some phases of adolescence, women are more perpetrators of physical and psychological violence than men,^24,25^ differing from the characteristics of intimate partner violence in adulthood.^26^ However, as for sexual violence, they are the main victims. Although reciprocal involvement in violence is pointed out as the most common pattern of ADV, the consequences are always worse for girls.^14,27,28^

Gender violence, such as early marriage, abusive sexual behavior, deprivation of work and inheritance and the impediment of family visits^29^ is trivialized or unrecognized in certain cultures. On the other hand, in cultures where this is recognized, ADV is less frequent, just as adolescents have more conservative sexual behavior and more egalitarian relationships.^30^

Racial discrimination, poverty and heterosexism are vulnerabilities found in the studies analyzed that were associated to ADV. The World Conference Against Racism held in Durban in 2001^31^ stresses the importance articulating between gender discrimination, racism, homophobia and class exploitation, common oppressions in the international globalization context. Alleyne-Green et al^32^ observed that ADV is particularly more likely in non-white girls.

A higher prevalence of ADV among non-whites was also found by Earnest et al^33^ in a study with 75,590 students. Some papers have shown that ADV is more prevalent in poverty contexts, especially in African Americans and among couples of sexual minority.^17,34,35^ The higher rate of violence in same-gender couples in some studies leads to a reflection on the weight of heterosexism and homophobia in the context of social vulnerability for the occurrence of ADV.^18,36,37^

**II - Circularity of Violence**

Violence in other contexts, such as in the family and in the neighborhood seems to be related to ADV,^15,38,39^ corroborating with the idea of circularity of violence brought by some authors.^40,41^ It is observed that structural violence of society that denies citizenship to some social groups is also related to community, intrafamily and interpersonal violence. It occurs in circular fashion in the diverse environments of adolescents’ socialization, dating relationships, family and friends.^2^ Patterns of social interaction learned throughout life may predict future violent behavior in intimate relationships. However, not all who are exposed to violence become aggressive adolescents.^19^ Cultural factors influence the emergence of ADV. A study conducted in United Kingdom and Spain revealed similar ADV rates in both countries. However, severe forms are more common in Spain, since in this country, milder expressions of violence are widely accepted as a normal event.^42^

Part of the studies of this review evidences an association between hostile treatment, anger and aggressive responses and ADV.^24^ Other studies have shown corporal punishment in the family and at school as ADV-associated factors.^14^ ADV-bullying was a frequent finding.^17,18^

The history of ill-treatment in childhood, witnessing intrafamily violence, poor care provided by parents and feeling insecure at school are found in several studies as strongly associated with ADV.^16,32-34^

Psychological distress may be the causal role in the relationship between domestic violence and victimization in adolescence dating.^43^ In addition, ADV can create a relationship pattern that persists in adulthood.^44^ The ADV rate is high among adolescents, estimated at 70% in a study with 299 mothers who were victims of domestic violence and their adolescent children. Adolescents reported perpetrating at least one of three dating abuse types.^15^ On the other hand, adolescents with a good family relationship, in which both parents are present, engage less in ADV and are less likely to tolerate or perpetrate some kind of violence in intimate relationships.^27,45^ ADV perpetration is also lower when adolescents have more propositional peer networks and neighborhood social control.^41^ The quality of the neighborhood is itself a contextual factor that can influence the emotional well-being of individuals. The bad neighborhood objective conditions, including poverty and instability of residence cause emotional distress that can increase the probability of ADV.^38^

Another context associated to ADV identified in papers was that of virtual violence.^46^ The popularity of using text messages, social media and Internet among adolescents can create opportunities for dating violence through virtual media. It includes attitudes such as monitoring, control, harassment or verbal and emotional abuse of a partner through technology, cell phones, threatening text or voice messages, or online publications of insulting content.

**III- ADV-associated health problems**

As mentioned in previous categories, the worst consequences of ADV are endured by females, and femicideis the most serious, the murder of women committed by men, typical of patriarchal regime, in which they are subjected to their control.^5^ Murder is one of the leading causes of death in young women and adolescents in the United States.^47^ However, none of the papers in this review focused on this serious and relevant issue. No studies were also found to relate ADV to suicide, another severe event in which a significant gender differential is evidenced, and women and sexual minorities are the most frequent victims.^48,49^

The consequences of ADV observed in the studies analyzed were low self-esteem, depressive symptoms, psychiatric disorders, drug abuse, risky sexual behavior and low academic performance.^18,32,50^ The more intense the violence, the greater the prevalence of severe psychiatric disorders common in adolescents. The association between psychiatric disorders and ADV varies with age, and the strength of the association decreased in magnitude with age.^51^ The acceptance of psychological abuse seems to mediate the association between ADV and psychiatric disorders such as depression, anxiety and hostility.^44^ The heavy consumption of alcoholic beverages and other drugs is associated with an increased perpetration of physical violence in dating.^41^ Copp^38^ identified that symptoms of anger and depression are found in cases of intimate partner violence associated with the disadvantageous neighborhood.

ADV is related to the use of chemical substances.^52^ This association is found when both partners are drug users, when only one is a drug user and with different types of drugs (alcoholic beverages, marijuana).^53^ In some studies, there was an intersection between ADV and substance use as a means of dealing with the disruption of the love relationship.^54^

**Final Considerations**

This review evidences that the phenomenon of ADV is complex since it involves multiple causes of an individual, social and cultural nature that require intersectoral actions to address it. The revised studies further clarify the potential health impairments resulting from ADV. However, it is worth highlighting the lack of research on homicides of adolescent and young women that could reveal other relevant angles of the problem to public health. The main recommendation of this review is the urgent and essential need of preventive actions that focus on the deconstruction of current cultural patterns of gender based on the revival of its history, in order to support emancipatory and liberating pedagogical approaches to be implemented early in schools, involving families and the community.
